# Resolving Secondary Infertility: A Case Report of the Successful Treatment of Sperm DNA Fragmentation Through the Physiological Intracytoplasmic Sperm Injection Method

**DOI:** 10.7759/cureus.53682

**Published:** 2024-02-06

**Authors:** Priyal Tilak, Pranita A Bawaskar, Ankit Badge, Mangesh Kohale, Jagadish G Makade, Nancy Nair

**Affiliations:** 1 Clinical Embryology, School of Allied Health Sciences, Datta Meghe Institute of Higher Education and Research, Nagpur, IND; 2 Microbiology, Datta Meghe Medical College, Datta Meghe Institute of Higher Education and Research, Nagpur, IND; 3 Pathology, Datta Meghe Medical College, Datta Meghe Institute of Higher Education and Research, Nagpur, IND; 4 Community Medicine, Datta Meghe Medical College, Datta Meghe Institute of Higher Education and Research, Nagpur, IND

**Keywords:** hyaluronic acid binding capacity, sperm concentration, physiological intracytoplasmic sperm injection, secondary infertility, sperm dna fragmentation

## Abstract

This case report presents an illustrative account of a couple experiencing secondary infertility attributed to the fragmentation of DNA in sperm. Secondary infertility, the inability to conceive after having previously successfully conceived a child, can be due to various factors, including male infertility issues. Sperm DNA fragmentation (SDF) has emerged as a major factor influencing male fertility, resulting in poor embryo development and lower pregnancy rates. This case is about the use of advanced assisted reproductive technologies, specifically physiological intracytoplasmic sperm injection (PICSI) and intracytoplasmic sperm injection (ICSI), to treat secondary infertility caused by fragmentation of sperm DNA. PICSI enables the identification and selection of spermatozoa with optimal functional integrity using hyaluronan, a natural binding substance. Preparing a PICSI dish requires skill and precision. Sperm exhibiting a high DNA fragmentation index were excluded from the selection process to enhance embryo development potential. The couple underwent controlled ovarian stimulation, egg retrieval, and ICSI with PICSI. The treatment resulted in the successful conception of a singleton pregnancy. Subsequent prenatal monitoring indicated a healthy pregnancy progression, ultimately culminating in the delivery of a healthy baby girl at term. This case report highlights the efficacy of integrating PICSI as a sperm selection method preceding ICSI, specifically in cases of secondary infertility related to SDF. Further research and larger-scale studies are warranted to approve the findings of this case report and establish the broader applicability of this treatment approach.

## Introduction

Couples seeking infertility treatment are becoming more prevalent, at around 17.5% worldwide [1]. When the establishment of a clinical pregnancy is not possible after 12 months of consistent, unprotected intercourse, it is called infertility. It is estimated that between 8% and 12% of couples in the reproductive age group are believed to be affected by this condition on a global scale [2]. In 35% of couples facing infertility, contributing factors can be attributed to male infertility. The main factors responsible for this condition include obesity, hypertension, diabetes, severe infections, genetic abnormalities, and external influences such as stress, smoking, and endocrine-disrupted chemicals. Compromised acrosomes, sperm plasma membranes, or chromatin can cause obstacles in embryo development, difficulties in uterine implantation, and recurrent miscarriages [3]. Men who require intracytoplasmic sperm injection (ICSI), such as those with oligo-asthenozoospermia, have a lower concentration and percentage of motile sperm in a sample [4] and frequently have sperm populations with increased aberration and compromised DNA integrity. Studies in embryos resulting from ICSI have been linked to a higher incidence of numerical and structural aberrations [5].

The ICSI technique is a common practice in assisted reproductive technology (ART). The selection of sperm for ICSI relies on a subjective evaluation of its morphology, which might not precisely pinpoint the highest-quality sperm. As an extra stage within the ICSI procedure, physiological intracytoplasmic sperm injection (PICSI) is a non-invasive method carried out on a sperm sample. The potential risks associated with ICSI also pertain to the use of PICSI. However, there are no additional recognized risks associated with PICSI, either for individuals who undergo fertility treatment or for children born through such treatment [6]. Hyaluronic acid (HA) receptors on plasma membranes are a maturation marker, and sperm that can bind immobilized HA in vitro are of higher quality [7]. The sperm selection process using the HA binding system can be applied in a laboratory setting and imitate the natural selection of male gametes cost-effectively. This approach improves the likelihood of successful pregnancies while also decreasing the occurrence of genetic complications [8].

## Case presentation

Couple history

This case report presents the experience of a couple who faced secondary infertility. They underwent treatment at the Wardha Test Tube Baby Center in Acharya Vinoba Bhave Rural Hospital, Sawangi (Meghe), Wardha. A female patient was 41 years old and her husband was 48 years old; they had been married for 20 years, and their first baby was conceived immediately after marriage at the age of 21 and 28, respectively, but they lost their child when he was 16 years old. They have been attempting to conceive for the last four years without success, despite achieving a successful pregnancy and birth. The patient reports regular menstrual cycles and no known medical conditions that could contribute to infertility. Now, they have visited the ART clinic for treatment. The male partner had habits of alcohol consumption and smoking, a history of hypertension for 10 years, no history of diabetes or thyroid in both partners, and no history of any surgery. The female partner has no addiction, no medical or past history, and they did not have a genetic abnormality in themselves or their family.

Clinical findings

Husband and wife appeared to be in physical and social well-being, without acute distress, and did not reveal any abnormalities. General health appears to be satisfactory, and there are no signs of endocrine or anatomical abnormalities. The seminal parameters of the male patient were taken, and the sperm count was measured at 19 million per milliliter (M/ml), with a total motility of the sperm of 30%, indicating the percentage of sperm capable of movement. Nonmotile sperm constitute 70% of the sample, while the pH level was recorded at 7.2, reflecting the acidity or alkalinity of the semen. The volume of the ejaculate was measured at 2.5 milliliters (ml). Morphological analysis reveals a significant concern, as 95% of sperm exhibit abnormal morphology, indicating structural abnormalities, while only 5% exhibit normal morphological characteristics. Table 1 shows the semen micro-examination report of the patient.

**Table 1:** Semen micro-examination report of the patient M/ml: million per millilitre, ml: millilitre, pH: potential of hydrogen

Semen parameters	Findings
Sperm count	19 M/ml
Total sperm motility	30%
Non-motile sperm	70%
pH	7.2
Volume	2.5 ml
Morphologically abnormal sperm	95%
Normal morphological sperm	5%

The patient was advised to undergo a sperm DNA fragmentation (SDF) test and a sperm vitality test, and the method used was a sperm chromatin dispersion (SCD) test. The distribution of sperm characteristics is categorized into different halo sizes. Among the evaluated sperm, 28 (14%) exhibit a large halo, 32 (16%) exhibit a medium-sized halo, and 45 (22.5%) each demonstrate a small halo and no halo at all. Additionally, 50 (25%) of the evaluated sperm population is classified as degraded. This comprehensive breakdown provides valuable information on the chromatin structure of sperm, shedding light on potential factors that may impact fertility. The results of SDF are shown in Table 2.

**Table 2:** SDF index report SCD: sperm chromatin dispersion, SDF: sperm DNA fragmentation

SCD	Sperms evaluated (n)	Percentage
Large halo	28	14%
Medium halo	32	16%
Small halo	45	22.5%
Without halo	45	22.5%
Degraded	50	25%

A blood test was conducted to assess female hormone levels, such as anti-mullerian hormone, follicle-stimulating hormone, and luteinizing hormone, which were found to be in the normal range, as shown below in Table 3.

**Table 3:** Results of the female hormonal profile AMH: anti-mullerian hormone, LH: luteinizing hormone, FSH: follicle-stimulating hormone, mIU/mL: milli-international units per milliliter, ng/mL: nanograms per milliliter

Hormonal profile	Reference limits	Findings
AMH	1.0-4.0 ng/ml	1.053 ng/ml
LH	2-15 mIU/ml	6.93 mIU/ml
FSH	3.5-12.5 mIU/ml	2.56 mIU/ml

Diagnosis

Based on clinical history, physical examination, semen analysis, and results of SDF tests, the patient has been diagnosed with secondary infertility caused by SDF, which refers to the presence of breakdown or damage in the DNA strands of sperm cells. This condition can negatively affect the ability of spermatozoa to fertilize an oocyte and lead to difficulty in achieving pregnancy.

Therapeutic intervention

The male patient was advised for an ICSI cycle with PICSI post-sperm separation by the density gradient method. The female partner underwent an ovarian stimulation protocol, and we administered short gonadotropin hormone-releasing hormone (GnRH) antagonists with regular monitoring. We used 5 mg of letrozole per day, and the GnRH antagonist was given 10000 IU for 36 hours before the ovum returned. On day 13, ovum pickup was done and retrieved; eight oocytes were MII and two were MI. PICSI was performed as per protocol; the procedure included the preparation of the sperm sample; the PICSI plate was prepared with a droplet of HA to mimic the natural selection of sperm; and after successful sperm selection, ICSI was performed.

**Figure 1:**
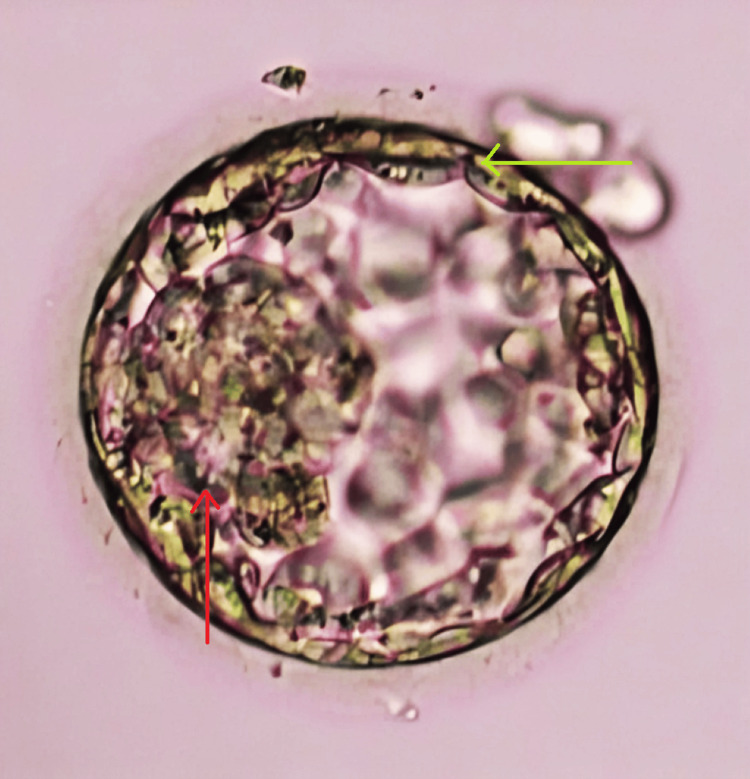
Day 5 blastocyst of grade 4AA transferred in the first cycle Inner cell mass (red arrow). Trophectoderm (green arrow). 4AA: good expansion and excellent inner cell mass and trophectoderm

**Figure 2:**
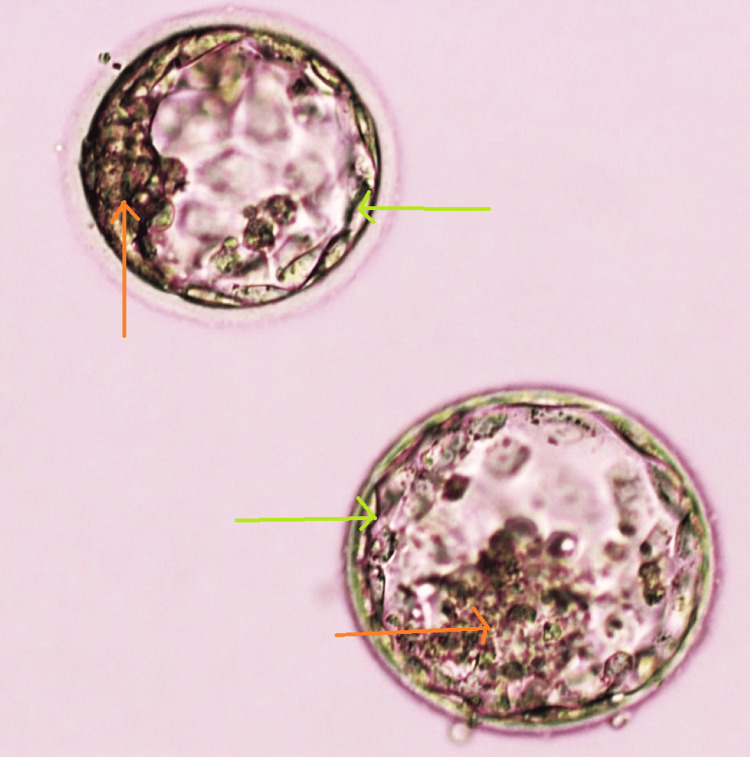
Day 5 blastocyst of grade 4BB and 4 BA transferred in the second cycle Inner cell mass (red arrow). Trophectoderm (green arrow). 4BB: expanded blastocyst with several loosely packed inner cell mass and trophectoderm cells. 4BA: expanded blastocyst with several loosely packed cells of inner cell mass and many cells organized in the epithelium of the trophectoderm

Five oocytes were fertilized, and three good-quality blastocysts of grades 4AA, 4BB, and 4BA were formed. All three blastocysts were cryopreserved in a 2+2 manner. After two months, a single frozen embryo of grade 4AA was selected for embryo transfer (ET) in the first cycle (Figure 1). On day 12, the 12th-day beta-human chorionic gonadotropin (β-hcg) level was negative. Later, we again transferred two frozen embryos of grade 4BB and 4 BA quality for ET in the second cycle (Figure 2), but this time β-hcg was positive, and after 37 weeks of gestation, a healthy baby was born.

Follow-up

After ET, the patient was instructed to avoid strenuous activity and heavy lifting and to rest. In both cycles, the patient also received calciferol sachets for calcium intake, prednisolone (5 mg), progesterone, calcium, multivitamins, and iron supplements. At follow-up, sonography revealed that the fetus had a normal growth rate and that the β-hcg level in the second cycle β-hcg level was positive (215 mIU/ml). The patient was advised to continue taking her medications. Coenzyme Q10 and zinc tablets were administered to a male patient once a day for three months.

## Discussion

DNA fragmentation plays an important role in the cause of male infertility. Compared to conventional sperm parameters, DNA fragmentation is a more reliable indicator of fertility potential [9]. In this case report, a significant contributor to infertility is the high DNA fragmentation. Men with elevated levels of DNA fragmentation experience a notable decrease in their likelihood of achieving conception by natural means or through assisted reproductive techniques such as IVF and intrauterine insemination [10]. Although the exact effects on sperm parameters are still being debated. Smoking, using tobacco products, and drinking alcohol negatively impact sperm parameters and DNA integrity. To establish the detrimental effects of alcohol and smoke on sperm morphology and DNA integrity, more sophisticated molecular research is needed. Examples of these studies include DNA methylation and gene polymorphism [11].

Anti-hypertensive drugs have a significant impact on sperm quality parameters and DNA integrity; in this case, our patient has been on spironolactone with a dose of 10 mg for the past 10 years, which could be a contributing factor to infertility [12]. Compared to men who did not take beta-blockers, men taking beta-blockers had lower volume, concentration, and motility. Lower sperm concentrations were observed in men who used calcium channel blockers. The volume of semen in men taking angiotensin receptor blockers was higher, but the concentration was lower. Lower volume and motility were observed in men taking angiotensin-converting enzyme (ACE) inhibitors [13].

We use physiological ICSI because it improves ICSI success rates by selecting sperm based on the oocyte's capacity to bind HA, although it takes skill to choose high-quality sperm. Spermatozoa chosen using the HA-binding method exhibit better internal and external qualities and a lower risk of aneuploidy or DNA fragmentation. Sperm chromatin is compacted, which protects and maintains DNA integrity, increasing its resistance to mutations and environmental stress. The failures may result in nuclear decompaction and de-condensations [14].

Compared to standard ICSI, PICSI did not produce a substantial increase in the term live birth rate. However, it is worth noting that there was a significant decrease in miscarriage rates among couples in the PICSI group [15]. The available evidence strongly suggests that the use of hyaluronan-based sperm selection mitigates the occurrence of miscarriages following ICSI. However, this reduction in miscarriage rates does not have a substantial impact on live birth rates. Despite this, there are still several potential advantages to using PICSI, which require further confirmation through additional research. One such advantage may be the potential for long-term health outcomes in offspring [16]. However, it is important to consider the cost-effectiveness of PICSI, as it is a highly expensive and time-consuming procedure. Other advanced methods of sperm selection include intracytoplasmic morphologically selected sperm injection, magnetic-activated cell sorting, and zeta potential sperm selection. Recent analyses have found that there is insufficient evidence to determine whether these techniques lead to better clinical outcomes, such as higher live birth rates [7,17].

## Conclusions

In this case report, secondary infertility caused by SDF was effectively treated using the PICSI technique. DNA fragmentation has been a major problem in male infertility, influencing fertilization rates and pregnancy outcomes. The application of advanced ART, such as PICSI, offers hope for improving sperm selection and potentially improving fertility treatment success rates. Although PICSI appears promising, more studies are required to fully understand its efficacy and effect on pregnancy and live birth rates. Couples facing SDF-related infertility should consult with a fertility specialist to explore the most suitable treatment options tailored to their unique needs and circumstances.
